# Comparative Genomic Analysis and Characterization of Two *Salmonella enterica* Serovar Enteritidis Isolates From Poultry With Notably Different Survival Abilities in Egg Whites

**DOI:** 10.3389/fmicb.2018.02111

**Published:** 2018-09-07

**Authors:** Yanyan Wang, Ben Jia, Xuebin Xu, Lida Zhang, Chaochun Wei, Hongyu Ou, Yan Cui, Chunlei Shi, Xianming Shi

**Affiliations:** ^1^MOST-USDA Joint Research Center for Food Safety, School of Agriculture and Biology and State Key Laboratory of Microbial Metabolism, Shanghai Jiao Tong University, Shanghai, China; ^2^School of Life Sciences and Biotechnology, Shanghai Jiao Tong University, Shanghai, China; ^3^Department of Microbiology, Shanghai Center for Disease Control and Prevention, Shanghai, China

**Keywords:** *Salmonella* Enteritidis, egg white, genome sequencing, genome comparison, survival

## Abstract

*Salmonella* *enterica* serovar Enteritidis (*Salmonella* Enteritidis) is a globally important foodborne pathogen, and the contaminated chicken eggs are the major source of salmonellosis in humans. *Salmonella* Enteritidis strains are differentially susceptible to the hostile environment of egg whites. Strains with superior survival ability in egg whites are more likely to contaminate eggs and consequently infect humans. However, the genetic basis for this phenotype is unclear. We characterized two *Salmonella* Enteritidis strains isolated from chicken meat that had similar genetic backgrounds but large differences in survival ability in egg whites. Although genome comparisons indicated that the gene content and genomic synteny were highly conserved, variations including six insertions or deletions (INDELs) and 70 single nucleotide polymorphisms (SNPs) were observed between the two genomes. Of these, 38 variations including four INDELs and 34 non-synonymous SNPs (nsSNP) were annotated to result in amino acid substitutions or INDELs in coding proteins. These variations were located in 38 genes involved in lysozyme inhibition, vitamin biosynthesis, cell division and DNA damage response, osmotic and oxidative protection, iron-related functions, cell envelope maintenance, amino acid and carbohydrate metabolism, antimicrobial resistance, and type III secretion system. We carried out allelic replacements for two nsSNPs in *bioC* (biotin synthesis) and *pliC* (lysozyme inhibition), and two INDELs in *ftsK* and *yqiJ* (DNA damage response) by homologous recombination, and these replacements did not alter the bacterial survival ability in egg whites. However, the bacterial survival ability in egg whites was reduced when deletion mutation of the genes *bioC* and *pliC* occurred. This study provides initial correlations between observed genotypes and phenotypes and serves as an important caveat for further functional studies.

## Introduction

*Salmonella* is a globally important foodborne bacterial pathogen. Poultry-derived products, particularly eggs and egg products, are the major vehicles for foodborne outbreaks of salmonellosis in humans (CDC, 2010, 2015, 2016, 2018a,b,c). Of more than 2600 serovars of *Salmonella, Salmonella enterica* serovar Enteritidis (*Salmonella* Enteritidis) is one of the leading serovars related with epidemics of salmonellosis (CDC, 2010, 2015; Martelli and Davies, 2012). *Salmonella* Enteritidis can contaminate eggs by two possible routes and may move into the egg white from the infected reproductive organs directly before eggshell formation and after laying via hen feces or storage environments (Guard-Petter, 2001; Messens et al., 2005; Martelli and Davies, 2012).

Egg white is a hostile environment for the survival of microorganisms including *Salmonella* Enteritidis (Guard-Petter, 2001; Gantois et al., 2009; Baron et al., 2016). Antibacterial properties of egg white include bacteriostatic and bactericidal components, an alkaline environment and nutritional restrictions including iron and biotin chelation (Guard-Petter, 2001; Gantois et al., 2009; Baron et al., 2016). In contrast, egg yolk is a suitable medium for growth of *Salmonella* Enteritidis (Bradshaw et al., 1990). Thus, an important precondition for prolonged contamination of an egg by *Salmonella* Enteritidis is the migration of bacterial cells from the egg white to the egg yolk. The rate of migration into the yolk is positively correlated with the concentration of *Salmonella* Enteritidis cells in the egg white (Braun and Fehlhaber, 1995). A greater cell number translates into a greater opportunity for contamination and for transfer to humans.

*Salmonella* Enteritidis exhibits higher survival ability in egg whites compared to other similar species or other *Salmonella* serovars (Clavijo et al., 2006; Gantois et al., 2008b; De Vylder et al., 2013). Remarkably, *Salmonella* Enteritidis strains differ in their survival abilities in egg whites, implying that some variants are better adapted to egg whites. The genomic variations responsible for these differences are an important issue to address but are unknown at present (Clavijo et al., 2006; Shah et al., 2012). DNA–DNA hybridization studies among *Salmonella* Enteritidis strains with significantly different phenotypes such as survival ability in egg whites or egg-contaminating ability could not distinguish these phenotypes genetically by this method (Morales et al., 2005; Yim et al., 2010; Shah et al., 2012). Whole-genome sequencing technology is a useful analytical tool to address these phenotypic differences and has a higher resolution.

In this study, we obtained two *Salmonella* Enteritidis isolates with similar genetic backgrounds but notably different survival rates in egg whites. Whole genome sequencing with high coverages was conducted to explore the genomic variations between the two strains. We analyzed potential genetic markers for differential survival abilities in egg whites. This study represents a new starting point for the exploration of the genomic evolution of *Salmonella* Enteritidis for egg contamination.

## Materials and Methods

### Bacterial Strains and Eggs

The *Salmonella* Enteritidis strains SJTUF10978 and SJTUF10984 were obtained from Shanghai Center for Disease Control and Prevention. They were isolated from chicken meat products from two different markets in Shanghai in 2010. The strains were preserved in -80°C and grown in Luria–Bertani (LB) broth (Oxoid, United Kingdom) at 37°C with shaking before use in assays. Unfertilized specific pathogen free (SPF) eggs were purchased from Beijing Merial Vital Laboratory Animal Technology Co., Ltd. (Beijing, China) and delivered to the lab within 3 days after laying. Thereafter, eggs were incubated at 37°C for an additional 3–5 days before use.

### Survival of *Salmonella* Enteritidis in Egg Whites

Survival rate of *Salmonella* Enteritidis in egg whites was measured according to a previously published method with minor modifications (Lu et al., 2003). We randomly selected eight SPF eggs and sterilized the shells with 75% ethanol before cracking. The egg white was separated from yolk and then mixed and homogenized for 10 min in a blender (easyMIX Lab Blender, AES Chemunex, Bruz, France). The mixed egg whites and normal saline (NS) were separately added into a 96-well plate at 200 μL each per well. One milliliter of overnight bacterial cultures was centrifuged at 5,000 ×*g* for 5 min and the pellets resuspended in 1 mL NS. The suspension was diluted with NS, and 30 μL of diluted cultures were then added to the egg white as well as to the NS in the 96-well plate in three replicates for each solution with final cell concentrations of approximately 2 × 10^3^ CFU/mL and mixed thoroughly. Portions (50 μL) of the bacteria–NS mixture from each well were plated on LB agar plates immediately (0 h). After 24 h incubation at 37°C, the whole volume of bacteria–egg whites mixture (230 μL) from each well was plated on LB agar plates (24 h). Colonies were enumerated after incubation at 37°C for 18 h. Survival rate of bacteria in egg whites was represented as ratio of the bacteria concentration in the 24-h sample compared to that of the 0-h sample.

Growth of *Salmonella* Enteritidis in LB used bacteria which was cultured and diluted as above. Thirty microliters of samples were added to 200 μL LB in 96-well plates in three replicates for each solution to a final concentration of approximately 2 × 10^3^ CFU/mL. Final concentrations were determined by plate counting as described above.

### Molecular Subtyping

Before genomic sequencing, strains SJTUF10978 and SJTUF10984 were analyzed by traditional typing methods. Pulsed-field gel electrophoresis (PFGE) was performed according to the procedures described in the standard operating procedure for PulseNet PFGE of *Escherichia coli* O157:H7, *E. coli* non-O157 (STEC), *Salmonella* serotypes, *Shigella sonnei*, and *Shigella flexneri*^1^. Multiple-locus variable number tandem repeat analysis (MLVA) was performed according to the procedures described in the PulseNet standard operating procedure for analysis of MLVA data of *Salmonella* Enteritidis in BioNumerics – Applied BioSystems Genetic Analyzer 3130/3500 data^2^. The MLVA profile was represented as a string of seven integers (VNTR1–VNTR2–VNTR8–VNTR6–VNTR5–VNTR3–VNTR9). The *Salmonella* sequence types (STs) were identified by uploading the chromosome sequences of the two strains to MLST version 1.8 (Larsen et al., 2012) with *S. enterica* as the MLST scheme.

### Genome Sequencing, Assembly, Finishing, and Annotation

Genomic DNA was prepared from overnight cultures using the DNeasy Blood and Tissue Kit as described by the manufacturer (Qiagen, Hilden, Germany). Libraries were constructed according to the TruSeq DNA sample preparation guide and sequenced on the Illumina HiSeq 2000 platform (Illumina, San Diego, CA, United States) to generate 2 × 100 bp paired-end sequencing reads. The reads were statistically analyzed and quality evaluated by FastQC^3^. Adapter sequences and low-quality reads with average quality scores <20 were removed from the data by Trimmomatic (Bolger et al., 2014). Reads that were less than 50 bp after trimming were also excluded from further genome assembly. Clean reads were assembled using the CLC Workbench 5.5 (Germantown, MD, United States). Contigs were then reordered based on comparison to the chromosome (GenBank accession no. NC_011294.1) and the plasmid pSEN (GenBank accession no. HG970000.1) of the *Salmonella* Enteritidis strain P125109 using progressiveMauve, version 2.3.1 (Darling et al., 2010). Gaps in the genomes were closed by sequencing PCR amplicons containing overlapping contigs on each side. The complete chromosome and plasmid sequences of SJTUF10978 and SJTUF10984 were submitted to the GenBank database and annotated with the Prokaryotic Genome Annotation Pipeline (PGAP; Version 3.2^4^) from the National Center for Biotechnology Information (NCBI).

### Genomic Characterization and Comparison

Variations between SJTUF10978 and SJTUF10984 were identified by the combination of two methods: one was mapping reads to the reference genome (NC_011294.1 and HG970000.1) using the Burrows-Wheeler Aligner (BWA, version 0.7.5a; Li and Durbin, 2009) and calling single nucleotide polymorphisms (SNPs) and small insertions or deletions (INDELs) with SAMtools (version 1.1; Mpileup/Bcftools; Li et al., 2009); another was comparing the complete genome sequences of SJTUF10978 and SJTUF10984 by progressiveMauve (Darling et al., 2010) and BLAST^5^. Variations were validated by mapping the sequencing reads back to the complete genomes of the two strains using Bowtie2 (Langmead and Salzberg, 2012). Genes carrying variations were named after their homologues reported in *Salmonella* or other bacterial species with close genetic relationships. For genes not mentioned in previous studies, the old locus tags (started by “*SEN*”) in the genome of *Salmonella* Enteritidis P125109 were used. The list of the variant genes with amino acid changes in the coding proteins were input into the PANTHER (Protein ANalysis THrough Evolutionary Relationships) database (Mi et al., 2017) for the overrepresentation test using *Salmonella* Typhimurium as the reference list. The significance of the result was assessed using Fisher’s Exact with FDR multiple test correction provided by this database. The non-synonymous SNPs (nsSNPs) were also analyzed by PROVEAN (Protein Variation Effect Analyzer; Choi et al., 2012; Choi and Chan, 2015), SIFT (Sim et al., 2012), PolyPhen-2 (Adzhubei et al., 2010), and SNAP2 (Hecht et al., 2015), to predict whether an amino acid substitution has an impact on the function of the coding protein. ResFinder 3.0 (Zankari et al., 2012) was used to screen antibiotic resistance genes of the two strains with a filter standard of 90% identity and 60% minimum length.

### Construction of Mutants and Growth Conditions

An in-frame deletion of the *bioC* gene in strains SJTUF10978 and SJTUF10984 were generated by overlap extension PCR (Ho et al., 1989). A 543 bp of fragment containing the upstream region of *bioC* and a 566-bp fragment containing the downstream region were generated from SJTUF10978 genomic DNA with primers *bioC*-UpF plus *bioC*-UpR and *bioC*-DnF plus *bioC*-DnR. A 10-bp overlap in the primers *bioC*-UpR and *bioC*-DnF allowed amplification of an 1109-bp product by a second PCR with primers *bioC*-UpF and *bioC*-DnR. The resulting PCR product, representing a deletion of the 76–708 bp region of the *bioC* gene, was then treated with T4 polynucleotide kinase and ligated into the suicide plasmid *pRE112* which had been digested with *Sma*I and dephosphorylated. The *pRE112* contained the counter-selectable marker *sacB1* as well as a chloramphenicol cassette. The recombinant plasmid was transformed into *E. coli* SM10 *λpir* and transformants (SM10-*pRE*Δ*bioC*) were recovered on LB agar supplemented with chloramphenicol. Plasmid *pRE*Δ*bioC* was transferred into the wide-type strains SJTUF10978 and SJTUF10984 by conjugation. The first crossover mutants with the *pRE*Δ*bioC* integrated into the chromosome were enriched in Selenite Cystine Broth (Beijing Land Bridge Technology, Beijing, China) containing chloramphenicol and screened on Xylose Lysine Deoxycholate Agar (Beijing Land Bridge Technology, Beijing, China) containing chloramphenicol. The first crossover mutants were then cultivated in LB with 10% (w/v) sucrose for 48 h to allow the second recombination and the loss of the suicide plasmid. The cultures were then plated on LB agar without NaCl but containing 10% (w/v) sucrose. Finally, the mutants (HΔ*bioC* and LΔ*bioC*, derivatives of SJTUF10978 and SJTUF10984, respectively) were negatively selected by chloramphenicol. This was confirmed by Sanger sequencing of PCR-amplified fragments. The in-frame deletion of the *pliC* gene in SJTUF10978 and SJTUF10984 (HΔ*pliC* and LΔ*pliC*, respectively) were generated using the same method as above. The double deletion of the *bioC* and *pliC* genes in SJTUF10978 was generated by introducing a *pliC* deletion in HΔ*bioC*. For the substitution of the single nucleotide in the position 433 from G to T in the *bioC* gene in SJTUF10978 (H*bioC*A145S), the method was almost the same as that in the deletion mutations described above except for the first step. That is, the recombinant was constructed by direct amplification of 433T along with the flanking regions of the *pliC* gene using primers *bioC*-UpF and *bioC*-DnR and genomic DNA of SJTUF10984 as template. The product was ligated into plasmid *pRE112*. Similarly, the first step for the substitution of the single nucleotide in the position 433 from T to G in the *bioC* gene in SJTUF10984 (L*bioC*S145A) was direct amplification of 433G along with the flanking regions of *bioC* with primers *bioC*-UpF and *bioC*-DnR and using the genomic DNA of SJTUF10978 as the template. The product was ligated to the plasmid *pRE112*. The methods for the exchange of the variant sequences contained within the three genes (*pliC, ftsK*, and *yqiJ*) between SJTUF10978 and SJTUF10984 were all similar to that for the *bioC* gene. The primers used in the construction of mutants are shown in **Supplementary Tables S3** and **S4**. Mutant information is presented in **Table 3**. In this study, the concentration of chloramphenicol was 35 μg/mL and the growth temperature for strains was 37°C.

## Results and Discussion

### Determination of Bacterial Survival Rate in Egg Whites

**FIGURE 1:**
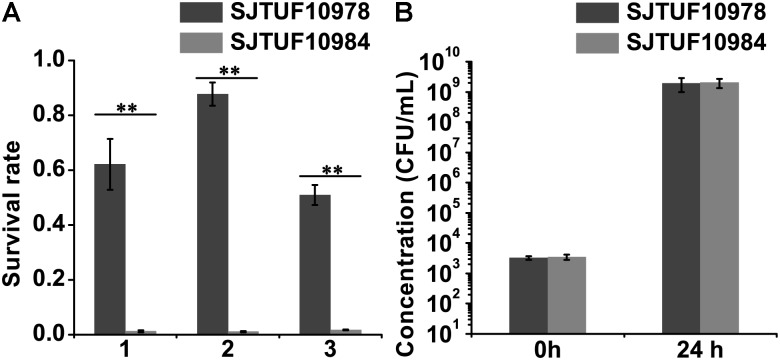
Survival of *Salmonella* Enteritidis strains SJTUF10978 and SJTUF10984 in egg whites. **(A)** The survival rate represents the ratio of surviving number of bacteria after 24-h incubation to the 0-h incubation in egg whites at 37°C. Results from three experiments performed in triplicate for each were shown. After incubation, SJTUF10978 displayed significantly higher survival rates than SJTUF10984 with 62.1:1.3, 87.7:1.1, and 50.9:1.8%, respectively, in three experiments using the egg whites from different patches of SPF eggs. **(B)** Growth in LB as control. The means ± standard deviations of results from three experiments performed in triplicate for each were shown. ^∗∗^*p* < 0.01, two-tailed *t*-test.

The survival rate of SJTUF10978 was significantly higher (52-fold on average) than SJTUF10984 after incubating in egg whites at 37°C for 24 h (**Figure 1A**). However, we found no growth differences in LB broth between the two isolates (**Figure 1B**). These data indicated that the higher survival ability of SJTUF10978 in egg whites was not due to general growth differences between the strains.

### General Genome Features

**FIGURE 2:**
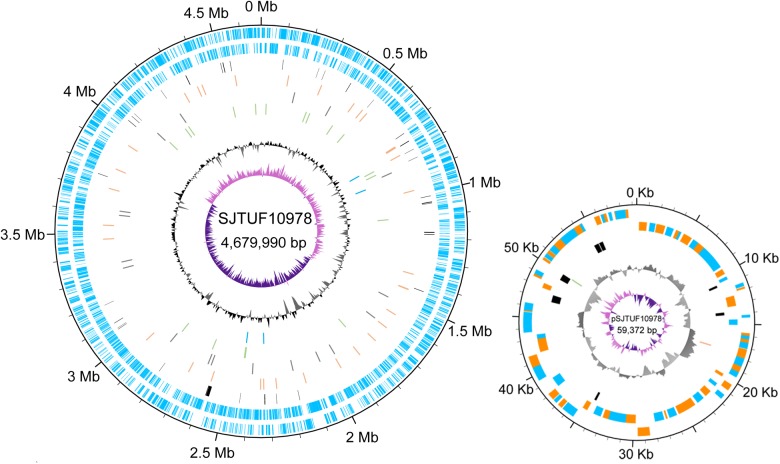
Circular map and genetic features of *Salmonella* Enteritidis strain SJTUF10978 relative to strain SJTUF10984. For the figure of the chromosome, light blue bars (first and second rings) indicate the coding sequences on the positive and negative strands, respectively. Orange, gray, green, and blue bars (fourth, fifth, sixth, and seventh rings) denote the non-synonymous SNPs, synonymous SNPs, intergenic SNPs, and INDELs between SJTUF10978 and SJTUF10984, respectively. For the figure of the plasmid of SJTUF10978, arcs in the first and second rings represent coding sequences on the positive and negative strands, respectively, and blue and orange were used to distinguish neighboring genes. The orange and green bars in the fourth ring indicate a non-synonymous SNP and an INDEL between plasmids of SJTUF10978 and SJTUF10984. For both figures, black bars (third ring) indicate pseudogenes and the black/gray and dark purple/light purple plots display GC content and GC skew ([G+C]/[G–C]), respectively (window size 10,000 bp and step size 200 bp for the chromosome, window size 500 bp, and step size 20 bp for the plasmid). The atlas was created using the Plotrix package (version 3.6-6; Lemon, 2006) within R (version 3.2.5; R Development Core Team, 2015).

High-throughput sequencing generated ∼1 GB of data with a 327-depth coverage for SJTUF10978 and a 234-depth coverage for SJTUF10984. The *de novo* assembly yielded 42 contigs with an N50 of 371,755 bp for SJTUF10978 and 44 contigs with an N50 of 277,421 bp for SJTUF10984. All contigs in these two genomes were successfully mapped to the reference genome with small variations in the alignments except for a deletion of a 5,998-bp fragment in both genomes compared to the reference genome of *Salmonella* Enteritidis strain P125109. Gap closure was accomplished and the complete genome sequence of SJTUF10978 was comprised of a 4,679,990-bp chromosome and a 59,372-bp plasmid. Similarly, the complete genome sequence of SJTUF10984 was comprised of a 4,679,791-bp chromosome and a 59,371-bp plasmid (**Figure 2** and **Table 1**). Both genomes have a GC content of 52.2%, comparable to the average GC content (52.1%) of *S. enterica* genomes reported in NCBI^6^. Both strains belong to the *Salmonella* ST 11 (ST11, alleles: *aroc*-5/*dnan*-2/*hemd*-3/*hisd*-7/*pure*-6/*suca*-6/*thra*-11). In addition, the same PFGE (*Xba*I) pattern and similar MLVA profiles: 5-4-1-10-11-3-3 in SJTUF10978 and 4-4-1-10-11-3-3 in SJTUF10984 were revealed before genome sequencing. None of the antimicrobial resistance genes in the Resfinder database (Zankari et al., 2012) was found in the two genomes.

**Table 1:** Genomic characteristics of two *Salmonella* Enteritidis strains with different survival abilities in egg whites.

	SJTUF10978	SJTUF10984
Chromosome	4,679,990 bp	4,679,791 bp
Plasmid	59,372 bp	59,371 bp
(G+C) %	52.20%	52.20%
Genes (total)	4,854	4,855
rRNAs	8, 7, 7 (5S, 16S, 23S)	8, 7, 7 (5S, 16S, 23S)
Pseudogenes	183	181
CRISPR Arrays	2	2
MLST	ST11	ST11
MLVA	5-4-1-10-11-3-3	4-4-1-10-11-3-3

The 5,998-bp deletion in the two genomes compared with the P125109 reference genome was further confirmed by checking the coverage of reads mapped onto the reference genome and comparisons using progressiveMauve (Darling et al., 2010). Interestingly, this deletion was identified in only 15 *Salmonella* genomes by the Microbial Genomes BLAST tools in NCBI (April 18, 2018). These strains were all identified as the Enteritidis serovar by submitting their genome sequences to the SeqSero web server (Zhang et al., 2015) although the NZ_CP019383.1 was labeled as *Salmonella* Typhimurium by the data submitter. Of these, 12 strains were isolated from Asia (five from China, five from South Korea, and two from Thailand) and implied that the evolutionary similarity between these 12 isolates and the two isolates in our study might be due to geographical proximity.

The 5,998-bp region in the reference genome NC_011294.1 encoded a DNA methyltransferase (part of *SEN4286*) and restriction endonucleases (*SEN4287, SEN_RS22295, SEN4291, SEN_RS22305*, and part of *SEN4292*). Notably, the *SEN4287* gene in strain SE2472 isolated from California in 1997 (Lu et al., 1999) was previously reported to have a positive effect on the survival rate of *Salmonella* Enteritidis in egg whites (Clavijo et al., 2006). As we were unable to compare the survival rate of SE2472 with the high survival strain SJTUF10978 in this study, we suspect that these strains may have comparable survival phenotypes in egg whites (Clavijo et al., 2006). If so, *Salmonella* Enteritidis strains from different areas in the world may have gone through a particularly adaptive evolution for survival in egg whites. However, if this was not the case, there must be genes other than *SEN4287* that caused differential survival ability in egg whites for the two closely genetic strains we used in the present study.

Whole genome alignment of the two genomes revealed that both the chromosomes and plasmids were collinear with strong consistency in gene arrangements and > 99% shared similarity in nucleotide sequences. Genome annotation identified 4,854 genes in the genome of SJTUF10978 and 4,855 genes in the genome of SJTUF10984 (**Table 1** and **Figure 2**). Moreover, there were 183 pseudogenes in the genome of SJTUF10978 and 181 in SJTUF10984 (**Table 1** and **Figure 2**). Among these, 180 were present in both genomes with identical sequences while four pseudogenes only existed in one of the two genomes (*sseL, yqiJ*, and *ratA* specific to SJTUF10978; *menH* specific to SJTUF10984; **Table 2** and **Figure 2**).

**Table 2:** INDELs and nsSNPs in coding sequences between SJTUF10978 and SJTUF10984.

Mutation type	Sequence change		Gene symbol	Predicted function
	Nucleotide	Amino acid		
**Lysozyme inhibition**				
nsSNP	C140T	A47V	*pliC*	Lysozyme inhibitor
**Vitamin biosynthesis**				
nsSNP	G1427A	G476D	*ushA*	Bifunctional UDP-sugar hydrolase
nsSNP	T433G	S145A	*bioC*	Biotin biosynthesis protein
nsSNP	T484C	X162Q	*menH*	2-succinyl-6-hydroxy-2,4-cyclohexadiene-1-carboxylate synthase
**Cell division and DNA damage response**				
INDEL	C2269_G2352ins	P757_Q784ins	*ftsK*	DNA translocase
INDEL	A23_T35del	frameshifted	*yqiJ*	Predicted inner membrane protein
nsSNP	C1249A	Q417K	*envC*	Murein hydrolase activator
**Osmotic and oxidative protection**				
INDEL	A157_G276ins	V53_T92ins	*ybiO*	Mechanosensitive channel protein
nsSNP	C847G	Q283E	*envZ*	Osmolarity sensor protein
nsSNP	A19T	I7F	*tpx*	Thioredoxin-dependent thiol peroxidase
nsSNP	G486T	L162F	*yccR*	Crp/Fnr family transcriptional regulator
**Iron-related functions**				
nsSNP	T562C	Y188H	*ydiU*	SELO (selenoprotein O) family protein
nsSNP	C907T	L303F	*sdaB*	L-serine deaminase II
nsSNP	A629G	Y210C	*dmsA*	Dimethyl sulfoxide reductase subunit A
nsSNP	G916A	V306I	*cfa*	Cyclopropane fatty acyl phospholipid synthase
nsSNP	G931A	V311I	*miaA*	tRNA dimethylallyltransferase
**Maintenance of cell envelope structure**				
nsSNP	T338C	L113P	*wecD*	TDP-fucosamine acetyltransferase
nsSNP	G113T	G38V	*fimI*	Major pilin protein
nsSNP	T535C	S179P	*stbE*	Fimbrial chaperone protein
nsSNP	A603G	I201M	*yejM*	Sulfatase
nsSNP	G775A	A259T	*ybiS*	L,D-transpeptidase
nsSNP	G355T	A119S	*ycdX*	Phosphatase / putative hydrolase
nsSNP	T79A	C27S	*yfaX*	Transcriptional regulator
nsSNP	T1342C	C448R	*cpsG*	Phosphomannomutase
**Amino acid and carbohydrate metabolism**				
nsSNP	T166A	C56S	*speD*	S-adenosylmethionine decarboxylase
nsSNP	A1290T	E430D	*hutH*	Histidine ammonia-lyase
nsSNP	G455A	S152N	*SEN2589*	Gluconolactonase
nsSNP	C614T	P205L	*dsdX*	D-serine permease
nsSNP	T382C	C128R	*ugpE*	Glycerol-3-phosphate transporter
nsSNP	T391C	W131R	*SEN4302*	Phosphotransferase enzyme II C component
nsSNP	A1583G	E528G	*actP*	Acetate permease
**Antimicrobial resistance**				
nsSNP	A229C	M77L	*sugE*	Multidrug efflux system protein
nsSNP	C287G	A96G	*ramR*	Transcriptional regulator
**Solitary genes**				
INDEL	C579ins	frameshifted	*sseL*	Deubiquitinase
nsSNP	T932A	L311H	*SEN1428*	Metal hydrolase
nsSNP	C1184T	A395V	*SEN1429*	Carboxylesterase
nsSNP	C1477T	Q493X	*ratA*	Outer membrane protein
nsSNP	T541G	S181A	*rsdB*	Resolvase

*Sequence changes from SJTUF10984 to SJTUF10978; “X” in column 3, premature stop codon*.

**Table 3:** Bacterial strains and plasmids used for mutant construction.

Bacterial strains/plasmid	Characteristics	Source
**Wild-type and mutants of SJTUF10978**		
H*wt*	*Salmonella* Enteritidis SJTUF10978, wide-type, high survival in egg whites	Chicken meat
HΔ*bioC*	In-frame deletion of *bioC* in SJTUF10978	This study
HΔ*pliC*	In-frame deletion of *pliC* in SJTUF10978	This study
HΔ*bioC*Δ*pliC*	Double in-frame deletion of *bioC* *pliC* in SJTUF10978	This study
H*bioC*A145S	G433T substitution in *bioC* in SJTUF10978	This study
H*pliC*V47A	T140C substitution in *pliC* in SJTUF10978	This study
H-mut-*ftsK*	Partial deletion (C2269_G2352) in *ftsK* in SJTUF10978	This study
H-mut-*yqiJ*	Gain-of-function mutation of *yqiJ* at position 23 (insertion of A23_T35 from SJTUF10984 *yqiJ*) in SJTUF10978	This study
**Wild-type and mutants of SJTUF10984**		
L*wt*	*Salmonella* Enteritidis SJTUF10984, wide-type, low survival in egg whites	Chicken meat
LΔ*bioC*	In-frame deletion of *bioC* in SJTUF10984	This study
LΔ*pliC*	In-frame deletion of *pliC* in SJTUF10984	This study
L*bioC*S145A	T433G substitution in *bioC* in SJTUF10984	This study
L*pliC*A47V	C140T substitution in *pliC* in SJTUF10984	This study
L-mut-*ftsK*	Insertion (C2269_G2352 from SJTUF10978 *ftsK*) at position 2269 in *ftsK* in SJTUF10984	This study
L-mut-*yqiJ*	Pseudogenization mutation of *yqiJ* (A23_T35 deletion) in SJTUF10984	This study
		
SM10 *λpir*	*Escherichia coli, thi thr-1 leu6 proA2 his-4 arg E2 lacY1 galK2, ara14 xyl5 supE44, λpir*	*(Rubires et al., 1997)*
*pRE112*	*pGP704* suicide plasmid, *pir* dependent, *oriT, oriV, sacB*, Cm^r^	(Edwards et al., 1998)

### Genetic Variations Between SJTUF10978 and SJTUF10984

A complete genome comparison revealed 76 variations including six INDELs and 70 SNPs between SJTUF10978 and SJTUF10984 (**Supplementary Table S1** and **Figure 2**). Four INDELs were located in coding regions in the chromosome and two were located in intergenic regions in the chromosome and the plasmid respectively. Of 70 SNPs observed in the two genomes, 34 were non-synonymous, 18 synonymous, and 18 intergenic. The coverage (average is shown for the INDELs) of the sequencing reads mapped to each variable site ranged from 88 to 465 (**Supplementary Table S1**). None of the synonymous and intergenic SNPs nor the two intergenic INDELs were considered. However, we cannot rule out the possibility that alterations of codon usage, regulatory sequences, or small RNAs have phenotypic effects.

The 38 variants, including 34 nsSNPs and four INDELs, were identified in the coding regions of 38 genes. Using these 38 genes as input, no gene was identified to be significantly overrepresented by the overrepresentation test in the PANTHER database (Mi et al., 2017). Manual functional analysis showed that these variant genes were involved in lysozyme inhibition, vitamin biosynthesis, cell division and DNA damage response, osmotic and oxidative protection, iron-related functions, maintenance of cell envelope structure, amino acid and carbohydrate metabolism, antimicrobial resistance, and type III secretion system (T3SS).

#### The SNP in *pliC* Is Not Responsible for the Differential Survival Ability in Egg Whites

**FIGURE 3:**
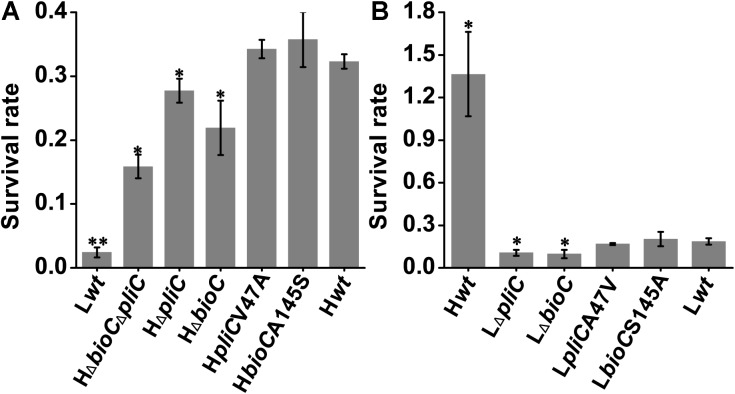
Survival of *bioC* and *pliC* mutants in egg whites. The survival rates of bacteria represented as the ratio of the concentration of bacteria at 24-h/0-h samples incubated at 37°C with starting inocula of approximately **(A)** 2 × 10^3^ and **(B)** 2 × 10^4^ CFU/mL (the initial concentration increased by an order of magnitude here because the survival rates of SJTUF10984 and its mutants in egg whites were too low to distinguish between each other when the inoculum concentration was 2 × 10^3^ CFU/mL). Survival rate is shown as the mean ± standard deviation. Experiments were repeated three times and results from one representative experiment were presented. ^∗^*p* < 0.05 and ^∗∗^*p* < 0.01, two-tailed *t*-test.

A missense variation (C140T, A47V) was found in the *pliC* gene in the genome comparisons (**Table 2**). This gene encodes a periplasmic lysozyme inhibitor of c-type lysozyme that is abundant in egg whites. C-type lysozyme is bacteriolytic and protects eggs against bacterial penetration. The probable antibacterial mechanism of this protein is peptidoglycan hydrolysis rendering the bacterial cell susceptible to osmotic lysis, or disturbing bacterial membrane structure due to the cationic and hydrophobic properties of this protein (Pellegrini et al., 2000; Ibrahim et al., 2001; Callewaert et al., 2008). In response, *Salmonella* Enteritidis is equipped with two kinds of lysozyme inhibitors, a periplasmic lysozyme inhibitor of c-type lysozyme (PliC) and a membrane-bound lysozyme inhibitor of c-type lysozyme (MliC; Callewaert et al., 2008). The nsSNP in *pliC* was predicted *in silico* to be deleterious for the coding protein (**Supplementary Table S2**). When we exchanged the SNP alleles in the *pliC* gene between the two strains, we found no significant changes of survival ability in egg whites (**Figure 3**). However, *pliC* gene deletion did result in decreased survival ability in egg whites for both isolates (**Figure 3**). This result was consistent with a *Salmonella* Enteritidis *pliC* knockout mutant that lost its *in vitro* inhibitory activity against lysozyme and showed increased lysozyme sensitivity (Callewaert et al., 2008).

#### Variant Genes Involved in Vitamin Biosynthesis

In this study, nsSNPs were found in three genes encoding enzymes for vitamins biosynthesis. They were in the *menH* (premature stop codon), *bioC* (missense variation), and *ushA* (missense variation) genes involved in the biosynthesis of menaquinone, biotin, and nicotinamide, respectively. Vitamins are important enzymatic cofactors used in basic metabolism (Begley et al., 2001; Beckett, 2007; Jiang et al., 2008). The *menH* gene encodes a provisional 2-succinyl-6-hydroxy-2,4-cyclohexadiene-1-carboxylate synthase involved in menaquinone biosynthesis (Jiang et al., 2008). This gene had evolved into a pseudogene in the low survival strain SJTUF10984. Interestingly, this genotype could not be found in the NCBI database suggesting that the degradation of the *menH* gene may have a non-negligible effect on the phenotype of this isolate. Moreover, the menaquinone content in egg white is low (0.9 μg/100 g; Schurgers and Vermeer, 2000). Based on these observations, it was suggested that *menH* might be involved in survival in egg whites if menaquinone was growth limiting.

The malonyl-CoA O-methyltransferase encoding gene *bioC* is involved in biotin synthesis. This biological process refers to a series of enzymes encoded by the *bioBFCD* operon and the *bioA* and *bioH* genes (Beckett, 2007). Avidin is an extremely high affinity biotin binding protein and makes egg whites biotin limiting (Baron et al., 2016). The biotin synthase gene *bioB* is required for the survival of *Salmonella* Enteritidis in egg whites (Raspoet et al., 2014). Moreover, *bioA, B, C, D*, and *F* are transcriptionally upregulated in *Salmonella* Enteritidis during growth in whole egg or egg white model medium (Jakociune et al., 2016; Baron et al., 2017). In our study, the single nucleotide variation (T433G, S145A) in the *bioC* gene was predicted to be deleterious *in silico* and suggested that this nsSNP had drastic functional consequences (**Supplementary Table S2**). Moreover, we found this same genotype in seven sequences in the NCBI database as in SJTUF10978, but 820 were identical to SJTUF10984. This indicates the particularity of BioC 145A in SJTUF10978 evolution.

To determine whether the nsSNP in *bioC* resulted in differential survival abilities in egg whites, the SNP alleles in *bioC* were exchanged between SJTUF10978 and SJTUF10984. However, we found no significant changes of survival rate (*p* > 0.05) in egg whites (**Figure 3**). However, *bioC* null mutants of SJTUF10978 and SJTUF10984 exhibited decreased survival rates in comparison with their respective parent strains (**Figure 3**). This demonstrates that *bioC* plays an important role in the survival of *Salmonella* Enteritidis in egg whites.

We also found growth defects when these two mutants were cultured in M9 minimal medium (data not shown). This is consistent with a growth deficiency for the *E. coli* *bioC* null mutant in the same medium (Del Campillo-Campbell et al., 1967). In addition, *bioC* *pliC* double deletions made SJTUF10978 more sensitive to egg whites than either single deletions (**Figure 3A**). This supports the hypothesis that *Salmonella* Enteritidis has developed a synergetic strategy to cope with the multicomponent bactericidal effects in egg whites (Baron et al., 2016). However, the comprehensive mechanism is yet unclear. Based on the above, the *bio* operon plays an essential role in biotin synthesis to satisfy the growth metabolism demand of *Salmonella* Enteritidis when encountering the biotin-limited environment in egg whites.

It was unexpected that the SNPs in *bioC* and *pliC* did not alter the survival ability although deletions in the two genes reduced the survival ability of *Salmonella* Enteritidis in egg whites. In fact, the transcription of the two genes were upregulated in egg whites compared with normal medium (data to be published), which further confirmed the role of the two genes in the bacterial survival in egg whites. We thought that there were two possible reasons why the SNPs in the two genes had no effect on the survival ability of the two strains in egg whites. First, the amino acids encoded by codons containing the SNPs are probably not located in the region(s) playing a key role in protein function (conserved regions of PliC: 71–78 and 87–93 and putatively conserved methyltransferase domain of BioC: 47–138, pfam08241; Callewaert et al., 2012; Marchler-Bauer et al., 2017). Second, it might be the synergistic effect of multi-site variation that led to the survival difference of *Salmonella* Enteritidis in egg whites. The effect of one single site may be very weak and may even have been masked by experimental error.

#### Variant Genes Involved in Cell Division and DNA Damage Response

In SJTUF10984, we identified an 84-bp deletion in *ftsK* that was not present in SJTUF10978, resulting in a 28-amino acid deletion (**Table 2**). FtsK is a DNA translocase playing a key role in chromosome segregation and consists of an N-terminal transmembrane domain, a proline/glutamine rich linker, and a C-terminal P-loop ATPase motif (Iyer et al., 2004). The 28-amino acid deletion removed 2/9 tandem repeats in the linker domain, predicted by the Tandem Repeats Finder (Benson, 1999). Comparison of different FtsK proteins shows variability in the linker domain (Massey et al., 2006). The linker is necessary for full activity of chromosome dimer resolution in *E. coli* (Bigot et al., 2004). FtsK levels increase during cell division and in response to DNA damage (Diez et al., 1997, 2000; Wang and Lutkenhaus, 1998). In addition, *ftsK* expression is regulated by ppGpp of the stringent response during nutritional deprivation (Diez et al., 2000).

In the *yqiJ* gene of SJTUF10978, a 13-bp insertion introduced a premature stop codon (**Table 2**). Notably, we did not find any identical sequences for this gene from SJTUF10978 in the NCBI database. This gene encodes a putative transmembrane protein with unknown function but is predicted to be involved in the cellular response to DNA damage in *E. coli* (Khil and Camerini-Otero, 2002).

*Salmonella* Enteritidis suffers nutrient limitation and DNA damage during egg white exposure (Lu et al., 2003; Baron et al., 2016). In this context, it was of interest to see if the *ftsK* and *yqiJ* INDELs would lead to the differences of survival ability in egg whites. Therefore, the *ftsK* and *yqiJ* alleles were exchanged between the two strains but we found no significant change (*p* > 0.05) of survival ability in egg whites between the mutants and the wild type strains (data not shown). This essentially rules out a functional significance for the *ftsK* and *yqiJ* INDELs for the bacterial survival in egg whites. It is speculated that there may be no difference in DNA damage repair ability between the two strains.

The *envC* gene is also involved in cell division (Hara et al., 2002; Heidrich et al., 2002) and we identified an nsSNP in this gene between the two isolates. The *envC* gene encodes a murein hydrolase activator and its deletion results in the formation of long filaments and higher lysozyme sensitivity (Hara et al., 2002; Heidrich et al., 2002). The nsSNP in *envC* was predicted *in silico* to have no effect on the protein, implying a lack of relevance with the bacterial survival ability in egg whites (**Supplementary Table S2**).

#### Variant Genes in Response to Osmotic and Oxidative Stress

In SJTUF10984, an nsSNP and a 120-bp deletion were identified in the *envZ* and *ybiO* genes, respectively (**Table 2**). Proteins encoded by the two genes are necessary for the bacterial response to osmotic challenges (Aiba et al., 1989; Edwards et al., 2012). Moreover, the nsSNP in *envZ* was predicted to affect protein function using *in silico* analysis (**Supplementary Table S2**). To accommodate a wide range of hosts and natural environments, bacteria have developed mechanisms to cope with osmotic stress to maintain cellular homeostasis (Wood, 2015). The shift from other environments to egg whites would probably bring changes of osmotic pressure for *Salmonella* Enteritidis. EnvZ is a sensory histidine kinase in the two-component regulatory system EnvZ-OmpR involved in osmoregulation through transcriptional control of the porin genes *ompF* and *ompC* (Aiba et al., 1989; Forst et al., 1989; Lan and Igo, 1998). EnvZ-OmpR system is also an important regulator of global gene expression of *Salmonella* Enteritidis in the egg white model medium (Baron et al., 2017). The *ybiO* gene encodes a putative mechanosensitive channel involved in osmotic adaption that is activated by hypo-osmotic shock (Edwards et al., 2012). Similarly, in a previous study, another mechanosensitive channel gene (*SEN3892*) is shown to be involved in the survival of *Salmonella* Enteritidis in egg whites suggesting it may function in the hypo-osmotic environment of egg whites (Clavijo et al., 2006). Conversely, in another study, the osmolarity of egg whites is shown to be slightly lower than that of Tryptone Soy Broth and is thought to be isosmotic with the cytoplasm of *Salmonella* Enteritidis (Baron et al., 2016). Based on these observations, further analysis is needed to confirm whether the variations in *ybiO* and *envZ* are responsible for the differential survival of *Salmonella* Enteritidis strains in egg whites. Two missense variations (nsSNPs) were found in the thiol peroxidase gene *tpx* and the Crp/Fnr family transcriptional regulator gene *yccR* (**Table 2**). The two genes are involved in oxidative stress and nutrient deprivation responses, respectively.

#### Iron-Related Variant Genes

Five genes possessing single nsSNPs for each between the two isolates (**Table 2**) were predicted to either encode iron-containing proteins or be regulons of iron-related transcriptional regulators. These genes include the SELO (selenoprotein O) family protein gene *ydiU*, the L-serine deaminase II gene *sdaB*, the dimethyl sulfoxide reductase gene *dmsA*, the cyclopropane fatty acyl phospholipid synthase gene *cfa*, and the tRNA delta (2)-isopentenylpyrophosphate transferase gene *miaA*. Of these, *ydiU* is upregulated by the iron–sulfur cluster regulator IscR in *E. coli* (Giel et al., 2006; Dudkiewicz et al., 2012) and possibly involved in the iron limitation response (Outten et al., 2004; Giel et al., 2006; Wada et al., 2011). Both *dmsA* and *cfa* are regulated by Fur (ferric uptake regulator), a transcriptional regulator that controls the iron homeostasis in bacteria (Troxell et al., 2011). In addition, iron is a necessary component for DmsA and SdaB function (Su and Newman, 1991; Troxell et al., 2011). Similarly, transcriptional changes of *dmsA* and the L-serine deaminase I gene *sdaA* (similar function with *sdaB*) has been observed in *Salmonella* Enteritidis exposed to the egg white model medium (Baron et al., 2017). MiaA-catalyzed tRNA modifications are most likely involved in siderophore (enterobactin) biosynthesis (Buck and Griffiths, 1982).

Iron restriction is a key factor in the antimicrobial activities of egg whites (Guard-Petter, 2001; Gantois et al., 2009; Baron et al., 2016). In this study, although no sequence differences were found in genes directly encoding siderophores or transporters for iron acquisition, five variant genes were involved in the response to iron limitation. Moreover, deleterious effects were inferred for the nsSNPs in *ydiU, sdaB*, and *dmsA* by *in silico* analysis (**Supplementary Table S2**). This implies that our two strains possess differential adaptive capacity to iron restriction in egg whites.

#### Variant Genes Involved in the Maintenance of Cell Envelope Structure

We identified eight nsSNPs distributed in the genes *wecD, fimI, stbE, yejM, ybiS, ycdX, yfaX*, and *cpsG*, all of which were involved in the maintenance of cell envelope structure (**Table 2**). The *wecD* gene encodes a TDP-fucosamine acetyltransferase required in the synthesis of the enterobacterial common antigen, a glycolipid in the external leaflet of the outer membrane (Ramos-Morales et al., 2003). The *fimI* gene is located in the type I fimbrial operon and encodes the type I fimbrial protein subunit (Rossolini et al., 1993). The *stbE* gene is predicted to encode a fimbrial assembly chaperone (Lindberg et al., 1989). The *yejM* gene specifying an inner membrane protein with sulfatase/phosphatase activity is proven essential in *E. coli*. The truncated mutation of the YejM protein causes increased permeability of the outer membrane and reduced lipid A synthesis (De Lay and Cronan, 2008). The *ybiS* gene encodes a provisional L, D-transpeptidase that catalyzes the covalent anchoring of the Braun lipoprotein to the peptidoglycan in *E. coli* (Magnet et al., 2007). The Braun lipoprotein contributes to the integrity of the outer envelope structure by connecting the outer membrane to peptidoglycan (Braun, 1975). The *ycdX* gene is predicted to encode a phosphatase and its mutation in *E. coli* conferred a swarming defect (Inoue et al., 2007). The *yfaX* gene (alias *rhmR*) encodes a putative DNA-binding transcriptional regulator for the *rhm* operon involved in L-rhamnose metabolism (Rodionova et al., 2013). Rhamnose is one of four stoichiometric sugars in the O repeat unit of LPS and rhamnose content is positively correlated with the molecular mass of LPS. The latter is further associated with *Salmonella* Enteritidis host infection and egg contamination phenotypes (Guard-Petter et al., 1999; Guard-Bouldin et al., 2004). The *cpsG* gene specifying the phosphomannomutase is involved in capsular polysaccharide biosynthesis (Stevenson et al., 1991).

Functional prediction by *in silico* analysis of the effects of these eight nsSNPs indicated that six were deleterious (**Supplementary Table S2**). Egg white lysozyme targets peptidoglycan and its action would enhance outer membrane permeability (Callewaert et al., 2008). Moreover, LPS and flagellin are needed for *Salmonella* Enteritidis to respond to the bactericidal effects of egg whites (Cogan et al., 2004; Clavijo et al., 2006; Gantois et al., 2008a). Thus, we speculate that the genes in this category play roles in cell wall repair in face of lysozyme destruction (*yejM* and *ybiS*), and for the maintenance of cell wall integrity and full functions of cellular appendages (*wecD, fimI, stbE*, and *yfaX*) for bacterial survival in egg whites.

#### Variant Genes Responsible for Amino Acid and Carbohydrate Metabolism

Seven nsSNPs were found in genes involved in amino acid and carbohydrate metabolism. Three of these genes are the S-adenosylmethionine decarboxylase gene *speD* (Xie et al., 1989), the histidine ammonia-lyase gene *hutH* (Meiss et al., 1969), and the gluconolactonase gene *SEN2589*. We also identified four transporter genes (*dsdX, ugpE, SEN4302*, and *actP*) encoding a D-serine transporter (Anfora and Welch, 2006), a glycerol-3-phosphate transporter (Yang et al., 2009), a putative sugar PTS (phosphotransferase system) permease, and an acetate transporter (Gimenez et al., 2003), respectively (**Table 2**). Previous studies have reported that amino acid and carbohydrate metabolism is linked to the survival phenotype of *Salmonella* Enteritidis in egg whites determined by transposon mutant library screening (Clavijo et al., 2006) and microarray analysis (Baron et al., 2017). These studies suggested that the two isolates might have differential adaptabilities to the nutrient restriction in egg whites.

#### Variant Genes Involved in Antimicrobial Resistance

Two genes possessing nsSNPs were involved in antimicrobial resistance (**Table 2**). The *sugE* gene encodes for a small multidrug resistance transporter (Chung and Saier, 2002; He et al., 2011). The *ramR* gene encodes a transcriptional regulator that negatively regulates the expression of the AcrAB efflux system via *ramA*. This system is involved in resistance to antibiotics from multiple classes (Abouzeed et al., 2008; O’Regan et al., 2009). The analysis of antibiotic resistance to 10 common antibiotics was carried out for the two strains but we did not observe any resistance phenotypes (data not shown); we therefore do not know the roles of these two multidrug transporters in the two strains.

#### Solitary Genes

The 579C insertion in the *sseL* gene resulted in a premature stop in SJTUF10978 compared with SJTUF10984 (**Table 2**). This gene encodes a deubiquitinating enzyme and is a translocated effector of the type T3SS encoded within *Salmonella* pathogenicity island-2 (SPI2; Coombes et al., 2007). Studies on SseL (*Salmonella* secreted factor L) function focused on its deubiquitination activity when interfering with ubiquitination pathways in hosts. This enzyme has been identified as a host colonization and cytotoxicity factor and was also involved in the regulation of lipid metabolism in infected host cells (Rytkonen et al., 2007; Mesquita et al., 2012). Similarly, a T3SS encoded within SPI1 is previously identified as a necessary factor for the survival of *Salmonella* Enteritidis in egg whites (Clavijo et al., 2006), and is downregulated in the egg white model medium (Baron et al., 2017) and upregulated in whole eggs (Jakociune et al., 2016). These studies further implied that both T3SS systems not only have an important role in host invasion but may also have an association with the survival of *Salmonella* Enteritidis in egg whites. In addition, SJTUF10978 genotype for *sseL* was not found in the NCBI database.

Apart from *sseL*, pseudogenization of secreted effectors seems common in *S. enterica* serovars (McClelland et al., 2004; Nuccio and Baumler, 2014; Klemm and Dougan, 2016; Klemm et al., 2016; Carden et al., 2017). One of these studies found that loss of the T3SS effector gene *sseI* is linked to an increased systemic disease in human hosts (Carden et al., 2017). Thus, future investigations are needed to determine whether *sseL* degradation confers SJTUF10978 better survival in egg whites or in hens. Additionally, the *sseL* gene is a regulon of the two-component regulatory system SsrA-SsrB and *ssrA* transcription is regulated by EnvZ-OmpR (Coombes et al., 2007). The co-occurrence of the variations in *envZ* and *sseL* further indicates a possibility for the two genes to be involved in the survival of *Salmonella* Enteritidis in egg whites.

The gene *ratA* encodes a putative outer membrane protein with unknown function (Kingsley et al., 2003) and was present as a pseudogene in SJTUF10978 via a single nucleotide substitution introducing an internal stop codon (**Table 2**). The remaining three variant genes due to single nsSNPs were *SEN1428, SEN1429*, and the plasmid gene *rsdB*. These genes encoded a putative metal hydrolase, a carboxylesterase and a resolvase (Krause and Guiney, 1991), respectively (**Table 2**).

## Conclusion

In the present study, we conducted the genome sequencing and functional analysis for two *Salmonella* Enteritidis isolates with similar genetic backgrounds but very different survival abilities in egg whites. Whole-genome sequencing and comparative analysis revealed that variant genes were involved in lysozyme inhibition, vitamin biosynthesis, cell division and DNA damage response, osmotic and oxidative protection, iron-related functions, maintenance of cell envelope structure, amino acid and carbohydrate metabolism, antimicrobial resistance, and T3SS. These may deter the effects of lysozyme, osmotic imbalances, vitamin starvation, and iron limitation. The variations we found in 21 of these genes were inferred *in silico* to affect functions of the encoding proteins. However, we could not directly determine whether these changes were connected with functional changes. For example, when we modified the nsSNPs in *bioC* and *pliC*, and the INDELs in *ftsK* and *yqiJ*, we found no phenotypic conversion of the mutants. Nevertheless, deletion of *bioC* and *pliC* gene did have phenotypic consequences.

Although the current results have not connected any variation to the survival phenotype of the two strains in egg whites, we suspect this difference is a comprehensive effect of many variations. In view of this, we now have collected more *Salmonella* Enteritidis strains in our lab. The phenotypic and genotypic analyses on these strains will be carried out in future work, which will provide more insights to the survival mechanism of *Salmonella* Enteritidis in egg whites. In this study, the two *Salmonella* Enteritidis isolates could hardly be separated from each other by traditional molecular typing methods such as PFGE and MLVA although they had 76 variations including INDELs and SNPs between the two genomes. This reminds us to be cautious about newly discovered strains in disease traceability and hazard assessment.

## Data Availability

The complete genome and plasmid sequences of SJTUF10978 and SJTUF10984 for this study can be found in the GenBank database, https://www.ncbi.nlm.nih.gov/genbank/ (Accession No. CP015524 ∼ CP015527).

## Author Contributions

YW, LZ, CW, HO, CS, and XS designed the study. YW performed the experiments and wrote the manuscript. YW, BJ, and LZ analyzed the data. XX, YC, CS, and XS contributed reagents, materials, and strains. All authors reviewed the manuscript.

## Conflict of Interest Statement

The authors declare that the research was conducted in the absence of any commercial or financial relationships that could be construed as a potential conflict of interest.

## Footnotes

1 https://www.cdc.gov/pulsenet/pdf/ecoli-shigella-salmonella-pfge-protocol-508c.pdf

2 https://www.cdc.gov/pulsenet/pdf/salmonella-mlva-e-abi-508c.pdf

3 http://www.bioinformatics.babraham.ac.uk/projects/fastqc/

4 http://ncbi.nlm.nih.gov/genomes/static/Pipeline.html

5 https://blast.ncbi.nlm.nih.gov

6 https://www.ncbi.nlm.nih.gov/genome/152
